# iTRAQ-Based Comparative Proteomic Analysis of Adult *Schistosoma japonicum* from Water Buffalo and Yellow Cattle

**DOI:** 10.3389/fmicb.2018.00099

**Published:** 2018-02-06

**Authors:** Qi Zhai, Zhiqiang Fu, Yang Hong, Xingang Yu, Qian Han, Ke Lu, Hao Li, Xuefeng Dou, Chuangang Zhu, Jinming Liu, Jiaojiao Lin, Guoqing Li

**Affiliations:** ^1^College of Veterinary Medicine, South China Agricultural University, Guangzhou, China; ^2^Key Laboratory of Animal Parasitology of Ministry of Agriculture, Shanghai Veterinary Research Institute, Chinese Academy of Agricultural Sciences, Shanghai, China

**Keywords:** *Schistosoma japonicum*, adult worm, water buffalo, yellow cattle, iTRAQ

## Abstract

Schistosomiasis japonicum is one of the most severe zoonotic diseases in China. Water buffalo and yellow cattle are important reservoir hosts and the main transmission sources of *Schistosoma japonicum* in endemic areas. The susceptibility of these two hosts to schistosome infection is different, as water buffaloes are less susceptible to *S. japonicum* than yellow cattle. In this study, iTRAQ-coupled LC-MS/MS was applied to compare the protein expression profiles of adult schistosomes recovered from water buffalo with those of yellow cattle. A total of 131 differentially expressed proteins (DEPs) were identified, including 46 upregulated proteins and 85 downregulated proteins. The iTRAQ results were confirmed by Western blotting and quantitative real-time PCR. Further analysis indicated that these DEPs were primarily involved in protein synthesis, transcriptional regulation, protein proteolysis, cytoskeletal structure and oxidative stress response processes. The results revealed that some of the differential expression molecules may affect the development and survival of schistosomes in these two natural hosts. Of note, this study provides useful information for understanding the interplay between schistosomes and their final hosts.

## Introduction

Schistosomiasis is caused by infection with trematodes of the genus *Schistosoma* and affects approximately 260 million people worldwide (WHO, 2016). Among the six species of *Schistosoma, S. japonicum* is popular mainly in China, Philippines and small pockets of Indonesia. Schistosomiasis control in China has been remarkably successful, and this disease has been efficiently controlled and even eliminated in many areas (Li et al., 2016). However, there were still 77,194 cases of schistosomiasis in China by the end of 2015 (Zhang L. et al., 2016). Endemic areas of this disease are distributed in the lake, marshland, and mountainous region of China (Liu et al., 2016; Zhang S. Q. et al., 2016). The results of an epidemiological survey indicate that yellow cattle and water buffalo are the most important reservoir hosts and the main source of transmission for schistosomiasis in China (Wang et al., 2005; Liu et al., 2012), because these animal species graze freely in areas where it is endemic and may be frequently in contact with schistosome-infested water, the infected animals spread more eggs into the environment than human and other animal hosts (Van Dorssen et al., 2017). Also water buffalo and yellow cattle may pose great threat to human infection because of the constant and close contact with humans in agricultural areas. Therefore, the effective intervention of bovine schistosomiasis will greatly further the process of controlling the disease in China.

The susceptibility of different hosts to infection with schistosomes varies. In the high *Schistosoma mansoni* transmission areas, some residents are chronical infected, while some individuals are considered “Putative Resistants” who are constantly exposed to *S. mansoni* infection but remained egg-negative (Viana et al., 1995; Gaze et al., 2014). Of the rodents, the hamster is permissive to *S. mansoni* and *Schistosoma haematobium* infection (Draz et al., 2008; Tallima et al., 2015), whereas the rat is semi-permissive host to *S. mansoni*, in which the worms do not cause typical granuloma in liver and cannot develop well (Hsu et al., 1973; Khalife et al., 2000; Amaral et al., 2016), and the mice show lower permissiveness to *S. haematobium* infection along with schistosomula’s delay in migrating through the lungs and adult worms’ low recovery rate (Dean et al., 1996; Rheinberg et al., 1998). In contrast to the other *Schistosoma* species, *S. japonicum* is a true zoonotic parasite. There are 46 species of mammals which have been reported to be naturally infected with *S. japonicum*, such as rabbits, cats, horses, goats, dogs, cattle, rats, and pigs. The susceptibility of different hosts to *S. japonicum* was also varied. Water buffaloes, rats, horses, and pigs are less susceptible to infection than yellow cattle, goats, and rabbits (He et al., 2001). Representative observations have shown differences in worm recovery rate, egg production, pathogenicity and immunological responses to parasite infection between these hosts (Jiang et al., 2010; Peng et al., 2011; Yang et al., 2012b). Moreover, the parasite self-cure phenomenon has been observed in water buffaloes and pigs, where parasites establish but are then eliminated in a self-cure response (Li et al., 2014).

Previous studies from our research group using microarray and comparative proteomic analyses revealed that schistosomula from susceptible BALB/c mice, less susceptible Wistar rats, and resistant *Microtus fortis* voles had different expression profiles (Hong et al., 2011; Peng et al., 2011). Another study compared the gene expression profiles of *S. japonicum* from water buffaloes, mice, and rabbits, and identified a panel of differentially expressed genes in worms from different hosts (Liu et al., 2015). This study suggested that the data exhibiting in worms from laboratory animals were not completely available to the worms from natural host animals. Since water buffaloes and yellow cattle are the most important sources of transmission of schistosomiasis in China and have distinct susceptibilities to *S. japonicum* infection, the use of these natural hosts as experimental animal models in the investigation of the schistosome–host interaction may contribute to the identification of key molecules involved in worm development, and screening of vaccine candidates or new drug targets, and consequently lead to the effective control of schistosomiasis in China.

For better understanding the development mechanism of schistosome in their natural hosts, water buffaloes and yellow cattle, we have compared the gene expression profiles of schistosomes from these two hosts in our lab, several differential expression genes which may be important for the development and survival of schistosome were identified (Yang et al., 2012a). However, the difference in protein expression levels in schistosomes from these natural hosts remains unknown to date.

Proteomics is a powerful tool for the high-throughput characterization of proteins combined with mass spectrometry and bioinformatics. As a quantitative method, the isobaric tag for relative and absolute quantitation (iTRAQ), is more suitable and sensitive for the comparison and identification of differentially expressed proteins (DEPs) compared to 2-D gel electrophoresis (Wu et al., 2006). In this study, water buffalo and yellow cattle were infected with *S. japonicum* and the iTRAQ technique was used to detect and quantify DEPs in adult worms from these two natural hosts. Our results may provide valuable information for screening potential vaccine candidates or new drug targets and consequently for the control of schistosomiasis in natural hosts in endemic areas.

## Materials and Methods

### Ethics Statement

All animal-handling procedures complied with the guidelines of the Association for Assessment and Accreditation of Laboratory Animal Care International (AAALAC). The animal use protocol was approved by the Institutional Animal Care and Use Committee of the Shanghai Veterinary Research Institute, Chinese Academy of Agricultural Sciences, People’s Republic of China.

### Worm Collection

Male yellow cattle and male water buffaloes aged 15–18 months, free of parasitic helminth infections and other infectious diseases, were obtained from schistosome non-endemic areas and used for experimental infection. All animals were housed in covered pens and received adequate care. *S. japonicum* (Chinese mainland strain) was routinely maintained in *Oncomelamia hupensis* at the Shanghai Veterinary Research Institute, CAAS. Yellow cattle and water buffaloes were challenged percutaneously with *S. japonicum* cercariae, respectively, on the upper back using the cover glass method as described previously (Da’Dara et al., 2008). Adult worms were perfused from the hepatic portal vein of the animals at 8 weeks post-challenge and the worm recovery rate was calculated. The worms were manually washed thoroughly in PBS to remove residual host debris. The equal amounts of worms obtained from water buffalo and yellow cattle were pooled and designated as B and C group, respectively. Then the worms were stored in liquid nitrogen until use.

### Protein Extraction

STD buffer (pH 8.0) containing 4%SDS, 150 mM Tris-HCl, 1 mM DTT and 0.1% v/v protease inhibitor mixture (Merck) were added to the adult worm samples and homogenized using a Dounce homogenizer. The mixture was heated at 100°C for 5 min and then sonicated on ice. The crude extract was heated again at 100°C for 5 min. After centrifugation, the supernatants were collected and protein concentration was determined using the Bio-Rad protein assay kit (Bio-Rad Laboratories, United States). The protein samples were analyzed by SDS-PAGE and CBB staining, then samples suitable for subsequent analysis was determined.

### Protein Digestion and iTRAQ Labeling

Proteins were digested according to the filter-aided sample preparation procedure, as previously described (Wiśniewski et al., 2009). Briefly, 200 μg of protein for each sample was diluted in 200 μL of uric acid (UA) buffer (8 M Urea, 150 mM Tris-HCl pH 8.0) and transferred onto a 10-kDa ultrafiltration filter. DTT and other low molecular-weight components were removed by centrifugation at 14,000 × *g* for 15 min and an additional washing step with 200 μL of UA buffer. Then, 100 μL of 50 mM iodoacetamide in UA buffer was added and the reaction tubes were vortexed for 1 min at 600 rpm. The samples were incubated for 30 min in the dark and centrifuged at 14,000 × *g* for 10 min. The filters were washed three times with 100 μL UA buffer and then twice with 100 μL dissolution buffer (50 mM triethylammonium bicarbonate at pH 8.5). The protein suspensions were digested with 40 μL trypsin buffer (2 μg trypsin in 40 μL dissolution buffer) and vortexed for 1 min at 600 rpm. The samples were incubated at 37°C for 16–18 h and centrifuged at 14,000 × *g* for 10 min. The resulting peptides were collected and the filters were rinsed with 40 μL dissolution buffer.

Peptide concentration was determined at an OD of 280 nm. iTRAQ labeling was performed using the iTRAQ Reagent-8 plex Multiplex Kit according to the manufacturer’s instructions (Applied Biosystems). The proteins of worms from water buffalo were labeled with reagents 113, 114, 115, and 116 whereas the proteins of worms from yellow cattle were labeled with reagents 117, 118, 119, and 121. Four independent repetitions were made for each group sample.

### Strong Cation Exchange (SCX) Fractionation

The labeled peptides were pooled and fractionated on an AKTA Purifier 100 system (GE Healthcare) using a SCX column (PolyLCInc, Columbia, MD, United States). Briefly, the peptides were dried in a vacuum concentrator, dissolved in 2 mL of buffer A (10 mM KH_2_PO_4_ in 25% of ACN, pH 3.0) and loaded onto a 4.6 mm × 100 mm Polysulfoethyl column (5 μm, 200 A, PolyLC Inc.) at a flow rate of 1 mL/min. The peptides were eluted at a flow rate of 1 mL/min with a gradient of 0–10% buffer B (500 mM KCl, 10 mM KH_2_PO_4_ in 25% of ACN, pH 3.0) for 7 min, 10–20% buffer B for 10 min, 20–45% buffer B for 5 min, and 45–100% buffer B for 5 min. The eluted peptides were collected and desalted using an offline fraction collector. The collected fractions were combined into 15 pools, vacuum freeze-dried, and desalted on C_18_ cartridges (Sigma). All collected fractions were stored at –80°C until further analysis.

### LC-MS/MS Analysis

The samples were separated using an Easy-nLC in the nano HPLC system. The column was equilibrated with 95% buffer A (0.1% formic acid). Each dried peptide was resuspended in 10 μL buffer A and loaded onto a Thermo Scientific EASY column (2 cm × 100 μm, 5 μm C_18_) using an autosampler. The peptides were eluted onto an analytical Thermo Scientific EASY column (75 μm × 100 mm, 3 μm C_18_) at a flow rate of 300 nL/min and separated using a 160-min gradient elution. The gradient was 50 min in 0–35% buffer B (0.1% formic acid, 84% acetonitrile), 50 min in 35–100% buffer B, followed by maintenance in 100% buffer B for 60 min. The HPLC eluates were directly electrosprayed into the mass spectrometer.

MS data acquisition was performed by using Q-Exactive (Thermo Finnigan, United States). Positive ion detection mode was used. Full-scan MS spectra (*m/z* of 300–1800) were obtained at a resolution of 70,000, *m/z* of 200, maximum ion accumulation time of 10 ms, and AGC target value of 3e^6^. The number of scan ranges was 1 with a 40-s dynamic exclusion. MS_2_ scans were performed using high-energy collisional dissociation (HCD) activation type at a resolution of 17,500, *m/z* of 200, and isolation window of 2 *m/z*. The microscans number was 1; the normalized collision energy was 30 eV; the maximum allowed ion accumulation times were 60 ms; and the under-fill ratio was defined as 0.1%. Twenty fraction profiles were collected after a full scan.

### Protein Identification and Quantification

All LC-MS/MS data were combined and converted using Proteome Discoverer software version 1.4 (Thermo Fisher Scientific Inc., United States). The fragmentation spectra were searched using the Mascot software version 2.2 (Matrix Science, United States). The *S. japonicum* protein sequence data used in the search were retrieved from UniprotKB (16511 sequences, release time 20161203). The following search parameters were used: MS/MS ion search; trypsin was specified as the digestion enzyme with two max missed cleavage; monoisotopic mass values; fixed modifications of carbamidomethyl (C), iTRAQ8plex (N-term) and iTRAQ8plex (K); variable modification of oxidation (M); peptide mass tolerance at ±20 ppm; MS/MS tolerance at 0.1 Da; unrestricted protein mass. All reported data were based on false discovery rate (FDR) of less than 1% confidence for protein identification.

Differentially expressed proteins were analyzed for significant up- or downregulation using the software Proteome Discoverer version 1.4. The parameters were set as follows: use only unique peptide and normalize on protein median. Ratios were used to assess the fold-change of the abundant proteins of worms collected from water buffalo and yellow cattle and Student’s *t*-tests were used to evaluate the significant (*P*-value < 0.05). A fold-change (water buffalo/yellow cattle) higher than 1.5 (*P* < 0.05) or lower than 0.67 (*P* < 0.05) considering the average of four replicates was considered significantly upregulated or downregulated. To assess the reproducibility of the MS data, hierarchical cluster analysis on quantification of identified proteins from replicates was conducted using R with average linkage method and Euclidean distance.

### Bioinformatics Analysis of DEPs of Adult Worms from Water Buffalo and Yellow Cattle

Protein sequences were clustered using the CD-HIT program with default parameters (sequence identity ≥ 95%) (Li et al., 2001; Huang et al., 2010). Isoelectric point (pI) and molecular weight were calculated using the Pepstats program in EMBOSS. Functional analysis of DEPs was performed using gene ontology (GO) analysis, and the DEPs were categorized according to the biological process, molecular function, and cellular component (Conesa et al., 2005). The Kyoto Encyclopedia of Genes and Genomes (KEGG) pathway analysis was used to classify and group the DEPs (Kanehisa and Goto, 2000). Protein–protein interaction (PPI) networks were built using the database STRING version 10 (Szklarczyk et al., 2015).

### Validation by Western Blot Analysis

Two DEPs, *Sj*GST and *Sj*PDI, which were identified as upregulated in worms from water buffalo, were used to confirm the proteomic results by Western blotting. A pre-processed equivalent amount of protein was obtained from each sample, separated by 12% SDS-PAGE, and transferred to 0.45-μm pore size nitrocellulose membranes (Merck Millipore). The membranes were blocked overnight at 4°C with 5% non-fat milk in 0.05% Tween 20-PBS and incubated with monoclonal antibodies specific to mouse β-actin (Beyotime, China, 1:500 dilution), and mouse sera specific to *Sj*PDI or *Sj*GST (1:100), respectively. The membranes were washed three times and incubated with horseradish peroxidase (HRP)-conjugated goat anti-mouse IgG (Beyotime, China, 1:2000 dilution). Moreover, the samples were detected using an automatic chemiluminescence imaging analysis system (Tanon, China).

### Quantitative Real-Time Polymerase Chain Reaction (qRT-PCR) Analysis

The genes encoding eight DEPs were investigated at the transcriptional level by qRT-PCR. The total RNA was isolated from mixed parasite samples from water buffalo or yellow cattle using TRIZOL Reagent (Invitrogen, Carlsbad, CA, United States) and transcribed into cDNA using the PrimeScript RT reagent Kit (TaKaRa, Osaka, Japan) according to the manufacturer’s instructions. Nicotinamide adenine dinucleotide (NADH) was used as a reference gene (Gobert et al., 2009) and the primers used in this study are presented in **Table 1**. PCR amplification was conducted using the SYBR Premix Ex Taq^TM^ kit (TaKaRa) in an ABI 7500 Real-time System (Life Technologies). The relative expression level of the genes was calculated by the 2^-ΔΔCt^ method (Livak and Schmittgen, 2001).

**Table 1:** qRT-PCR primers and the comparison of the results between iTRAQ and qRT-PCR.

Uniprot ID	Primer (5′–3′)	Fold change in qRT-PCR	Fold change in iTRAQ
Q86EB6	FP: CACCATTACTTGATATTGAAGATT	1.36	1.74
	RP: TTAGGAAGGATGCCAGTC		
Q5BS32	FP: TCAGAAGTAGAAGAGACAT	0.62	0.56
	RP: GAATAAGACCACAATAATGATT		
Q5DDW4	FP: GAAGAGGTTACTGCTTGATA	5.91	4.05
	RP: TGATTGGACATCCGATTC		
C1LB69	FP: CGATGGATGTGCTGTAAT	1.56	2.03
	RP: ACTGGTCAACTTCATCAC		
C1LNS2	FP: AAGAAGATGTTAGCAATGAA	0.62	0.64
	RP: TCCTTATCCGTATCAACTT		
C1LHC4	FP: GTCCTTATTGTACTTGTTGTC	0.73^∗^	1.53
	RP: AAATCCCACCATCTTCTAAA		
C1LAT6	FP: TTATGTCGCAAGCAGATG	1.94^∗^	0.33
	RP: TTGTAGGTCTTAGCAAGTT		
C1LI02	FP: TGCTTACATTAGACACTTCTCT	0.50	0.48
	RP: TTGCGTTCACCAATCCTT		

*^∗^ Gene relative expression in schistosomes from water buffalo compared with those from yellow cattle has opposite expression regulation between qRT-PCR analysis and iTRAQ analysis.*

### Statistical Analysis

Student’s *t*-tests were used to evaluate deviations of protein quantification and worm recovery rate between two groups of worm samples by using SPSS for Windows version 22 (SPSS, Chicago, IL, United States), and *P*-values of less than 0.05 were considered statistically significant. In addition, the cluster analysis of protein quantification was performed in the R environment using gplots package. Fisher’s exact test was used to assess the KEGG enrichment analysis and *P*-values of less than 0.05 were considered significant.

## Results

### Adult Worm Recovery and Protein SDS-PAGE Analysis

**FIGURE 1:**
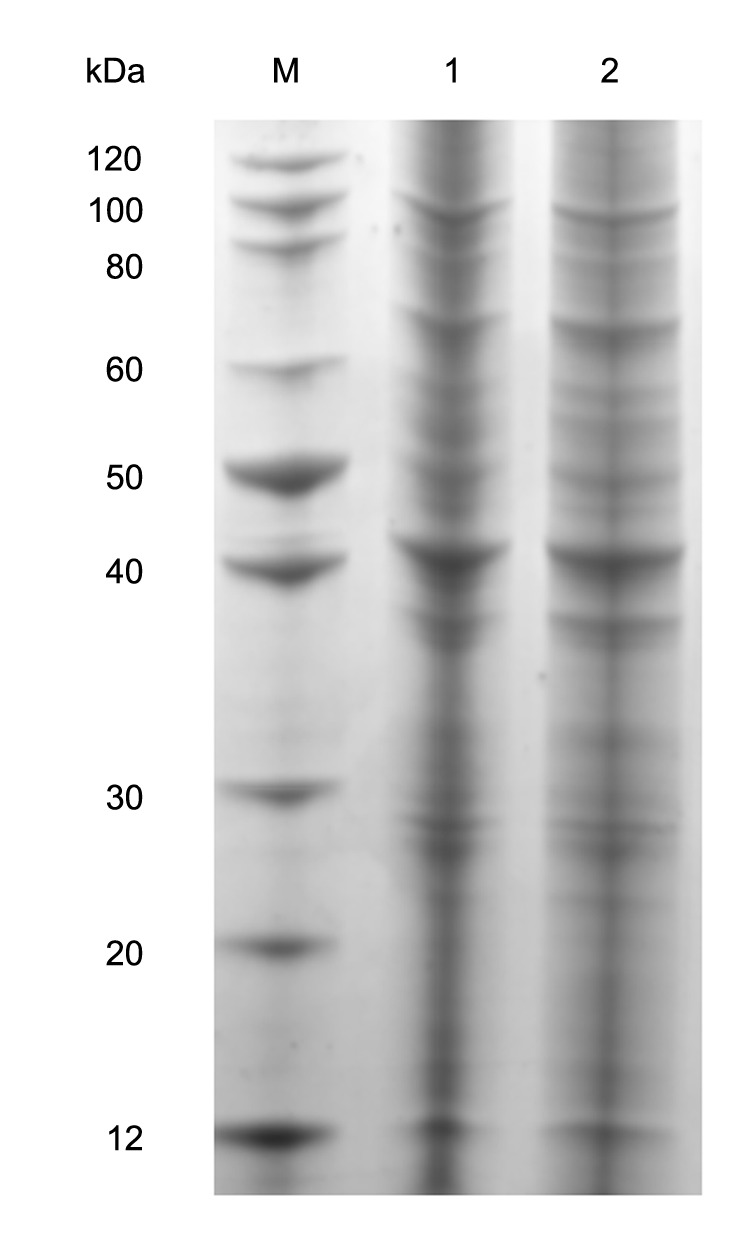
Separation of complete proteins of adult schistosomes from water buffalo and yellow cattle. 1: from yellow cattle; 2: from water buffalo.

The average worm recovery rate from water buffalo and yellow cattle was 10.8 and 68.8%, respectively (Supplementary Table 1). Each sample containing equivalent proteins was separated on 12% SDS-PAGE (**Figure 1**) and clearly distinct band patterns were visualized. The protein isolation was suitable for trypsin digestion and LC-MS/MS analysis.

### Global Proteomic Analysis of Schistosomes from Water Buffalo and Yellow Cattle

**FIGURE 2:**
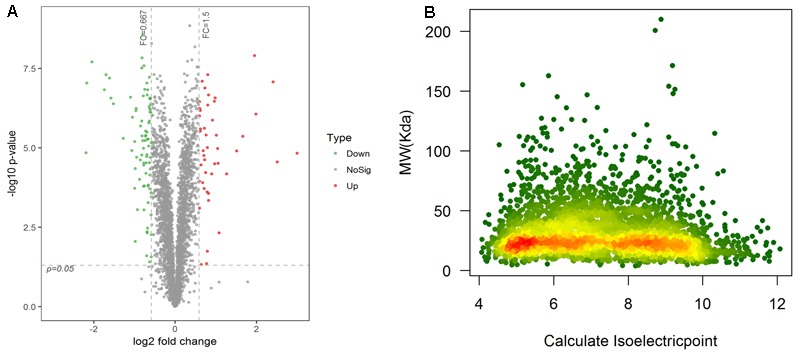
**(A)** Ratio distribution of all quantitative proteins. Differentially expressed protein marked in red or green (red indicates upregulation and green indicates downregulation). **(B)** Theoretical 2-D (pI, MW) distribution of identified DEPs.

A total of 18,288 unique peptides corresponding to 3,605 proteins were identified by iTRAQ and LC-MC/MC analysis. Using CD-HIT filtration with sequence identity ≥95%, 3,407 proteins were identified, of which 3,398 were quantified (**Table 2**). The reproducibility of the MS data were compared, which indicated that the two group protein samples separated into two main clusters (Supplementary Figure 1). Proteins with a statistically significant (*p* < 0.05) fold-change of 1.5 or greater were considered differentially expressed. Compared with schistosomes from yellow cattle, 131 proteins were found to be differentially expressed, of which 46 were upregulated and 85 were downregulated (**Figure 2A** and Supplementary Table 2). The molecular mass of DEPs ranged from 6.7 kDa to 113.8 kDa and the pI of DEPs ranged from 4.25 to 11.78 (**Figure 2B** and Supplementary Tables 3, 4). Ribosomes, transcription regulation-associated proteins, and cytoskeletal structure-associated proteins were the most abundant among the downregulated DEPs. Lysosomal proteins and oxidative stress response proteins were the most abundant among the upregulated DEPs. Some of the DEPs were listed in **Table 3**.

**Table 2:** Results of LC-MS/MS.

Type	Number
Total spectra	286182
Spectra matched peptide	69075
Peptide	20913
Unique peptide	18288
Protein identified	3407
Protein quantitated	3398

**Table 3:** Selected DEPs of adult *Schistosoma japonicum* worms from water buffalo and yellow cattle.

Accession	Description		Coverage (%)	Ratio (water buffalo vs. yellow cattle)
**Protein synthesis**				
C1LAT6	40S ribosomal protein S24		41.09	0.3264
C1LEQ5	Ribosomal protein L29		18.84	0.5832
C1LQX0	Ribosomal protein L35		29.66	0.6640
Q5BYE5	SJCHGC04807 protein		3.6	0.6195
C1LGX0	U3 small nucleolar RNA-associated protein 14		2.25	0.5854
C7TY97	Ribosomal protein L19		42.86	1.629
**Transcription regulation**		
Q5DCT2	U1 small nuclear ribonucleoprotein C		21.21	0.5021
Q5BSE3	Small nuclear ribonucleoprotein F		11.9	0.5128
Q5DGI6	SJCHGC04552 protein; similar to U4/U6 small nuclear ribonucleoprotein PRP4		2.46	0.5902
Q5C0I0	Spliceosome-associated protein, putative		4.6	0.6490
C1L4T5	Putative SAM pointed domain containing ETS transcription factor		6.22	0.6381
C1LK36	Single-stranded DNA-binding protein 3		1.78	0.5057
C1LEB4	IWS1 homolog		2.89	0.5014
Q5DET3	Histone H4		52.43	0.6408
C8CHI9	cyclophilin A		16.28	0.6306
Q5DDW4	SJCHGC09169 protein; similar to U4/U6.U5 tri-snRNP-associated protein 1		6.33	3.971
C1LRV3	Basic-leucine zipper (BZIP) transcription factor, domain-containing protein		2.16	2.40
**Cytoskeletal structure**		
B3W666	Tropomyosin		78.52	0.6579
C1LNS2	Tropomyosin-2		79.23	0.6441
C7TZG8	Myosin heavy chain		69.43	0.6254
O96398	Myosin		77.56	0.5812
Q5C296	SJCHGC01885 protein		78.89	0.6492
Q5DB55	Myosin regulatory light chain 2, smooth muscle minor isoform (G1)		68.69	0.6222
Q5DCQ8	Myosin alkali light chain 1		91.03	0.5867
Q5DCT5	SJCHGC09440 protein		34.59	0.6568
Q5DGG9	Troponin T		63.21	0.6499
Q5BS32	Dynein light chain roadblock		12.37	0.5555
C1LI02	Transmembrane protein 167 precursor		13.89	0.4772
C1LEX2	Inner nuclear membrane protein Man1 (LEM domain-containing protein 3)		7.13	0.6609
**Protein proteolysis**		
F6LHR8	Tetraspanin 2		27.44	1.742
C1LBS5	Cathepsin L, a		34.14	1.694
C1LAA5	Saposin B domain-containing protein		24.86	1.957
**Oxidation reduction**				
Q75UG3	Thioredoxin peroxidase-1		67.93	2.022
C1LNQ6	Protein disulfide-isomerase		65.92	1.656
C1LCI7	Glutathione *S*-transferase		75.36	1.655

*The protein accession numbers are from Uniprot (http://www.uniprot.org/uniprot/). The coverage was defined as the ratio (%) of the protein sequence covered by the matched peptides.*

### Bioinformatic Analysis of the DEPs

**FIGURE 3:**
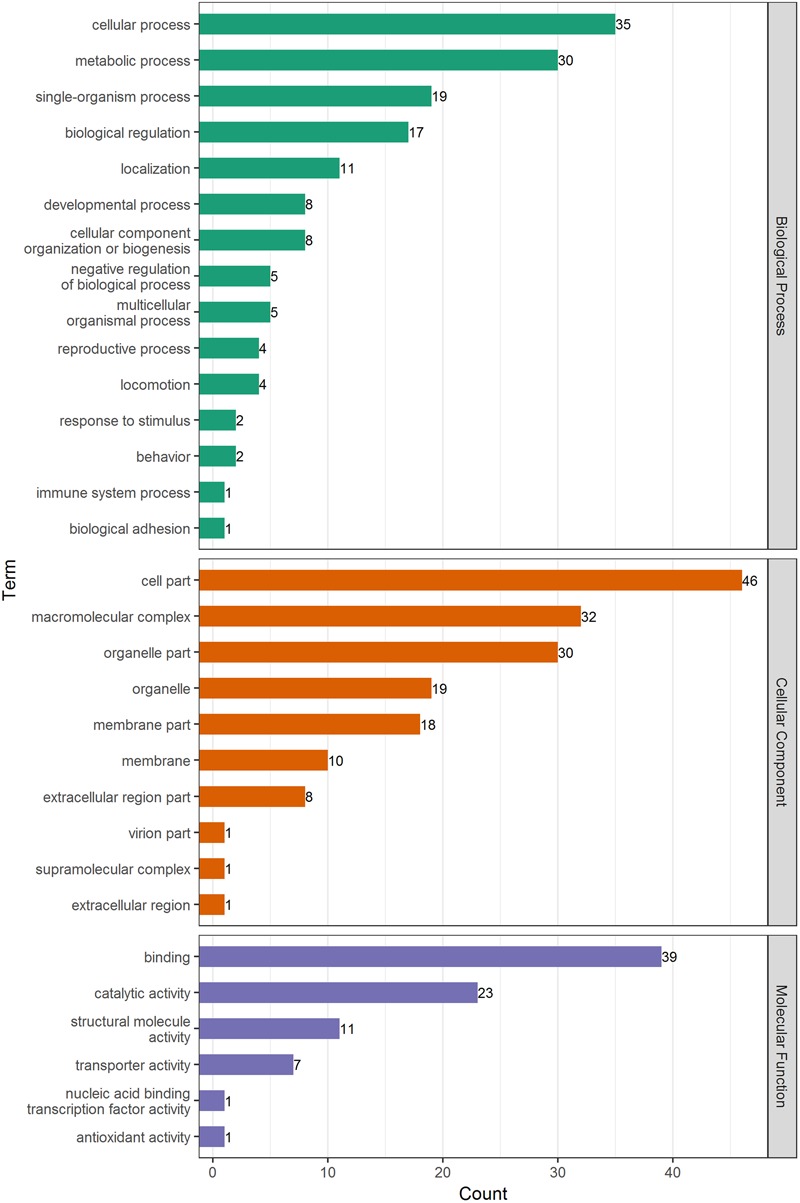
GO analysis of DEPs in adult schistosome from water buffalo and yellow cattle. Proteins were classified into three main categories: biological process, cellular component, and molecular function.

Gene ontology analysis was used to identify significantly enriched functional terms of DEPs (**Figure 3** and Supplementary Tables 3, 4). The results showed that DEPs were primarily involved in cellular, metabolic, and single-organism processes; biological regulation; localization; cellular component organization or biogenesis, and other biological processes. With regard to the cellular components, most DEPs were associated with cell composition, macromolecular complexes, organelles, membrane structure, and other components. Under the category of molecular function, most DEPs were correlated with binding, catalysis, transport, and structure, among others.

**FIGURE 4:**
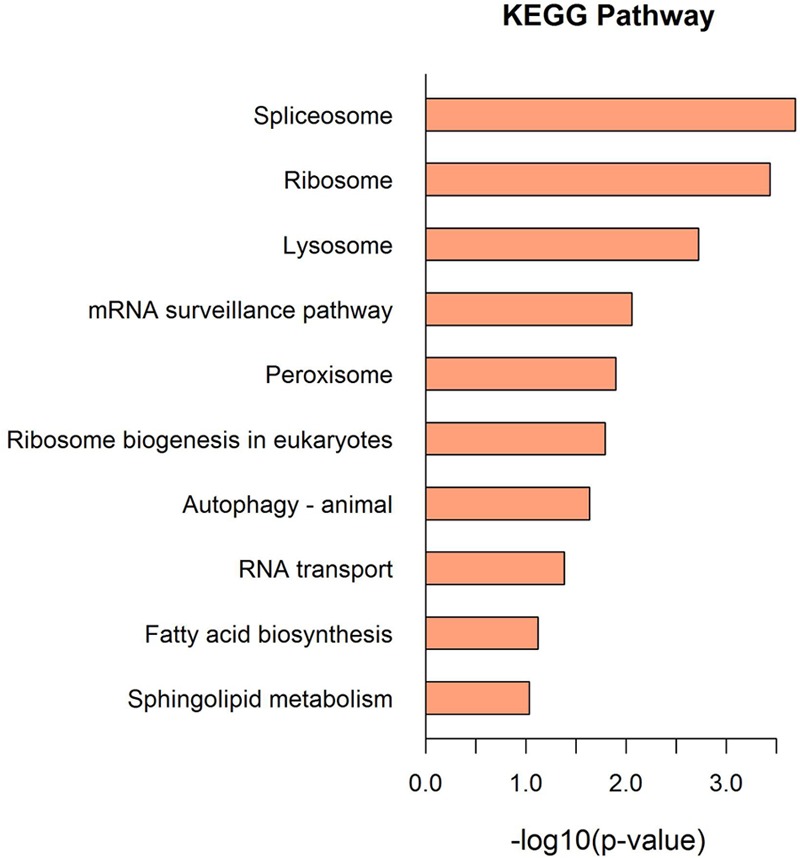
The KEGG pathway enrichment analysis of the DEPs. Top 10 enrichment in KEGG pathway maps of the DEPs. *P*-value was calculated using Fisher’s exact test.

The KEGG pathway and enrichment analysis indicated that the DEPs were highly enriched in transcription, translation, and transport and catabolic pathways, including spliceosome, ribosome, lysosome, mRNA surveillance, and peroxisome (**Figure 4** and Supplementary Tables 3, 4).

**FIGURE 5:**
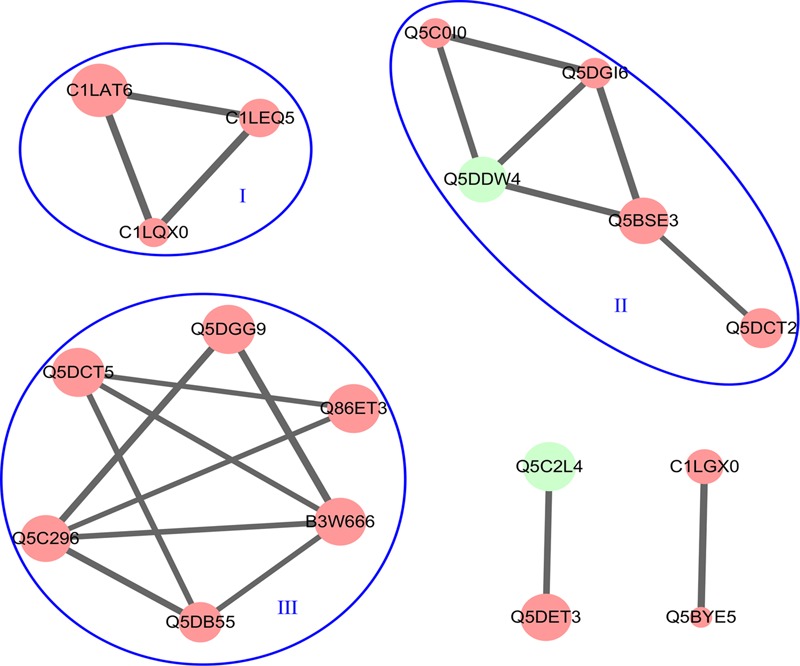
The PPI networks of all DEPs. The PPI networks were built using STRING version 10.0 with a high combined score >700. The node color indicates downregulated proteins (red) and upregulated proteins (green).

The PPI networks of DEPs were built and analyzed using STRING version 10. There were 18 nodes and 20 edges in the PPI network (**Figure 5**). Three distinct clusters with a high combined score were found among the downregulated proteins, and these clusters were associated with or involved in protein synthesis (I), transcription regulation (II), and cytoskeleton-associated proteins (III). No significant protein–protein interactions were observed among the upregulated proteins.

### Validation of the DEPs of Schistosomes from Water Buffalo and Yellow Cattle

**FIGURE 6:**
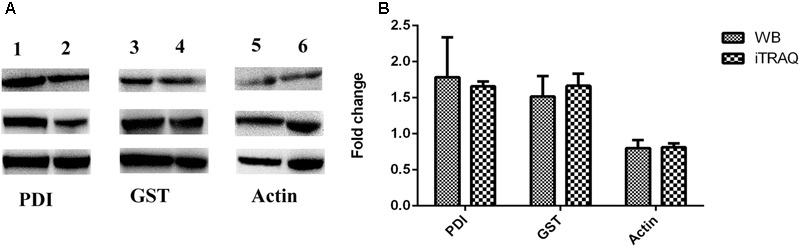
Confirmation of differentially expressed proteins by Western blotting. Each experiment was repeated three times. **(A)** Immunoblot analysis of proteins (*Sj*PDI, *Sj*GST, and actin) in adult schistosome from water buffalo and yellow cattle. Lane 1, *Sj*PDI of water buffalo group. Lane 2, *Sj*PDI of yellow cattle group. Lane 3, *Sj*GST of water buffalo group. Lane 4, *Sj*GST of yellow cattle group. Lane 5, actin of water buffalo group. Lane 6, actin of yellow cattle group. **(B)** WB ratios (water buffalo group/yellow cattle group) were consistent with those obtained by iTRAQ.

Three replicates of Western blot experiments were performed to confirm the iTRAQ results. The Western blot analysis indicated that two proteins, *Sj*GST and *Sj*PDI, were highly expressed in schistosomes infecting water buffaloes (**Figure 6** and Supplementary Figure 2), which was consistent with the results of iTRAQ, although there were slight differences in the mean values between the iTRAQ and Western blotting. These results further confirmed the changes in DEPs detected by iTRAQ.

### qRT-PCR Analysis of DEPs

Since the specific antibodies available for DEPs in our laboratory is limited, qRT-PCR was performed to further verify the reliability of the iTRAQ results at the transcriptional level. The mRNA transcription levels of eight DEPs were measured and six of them showed expression patterns similar to those of iTRAQ whereas the transcriptional pattern of the other two DEPs differed from the protein expression pattern (**Table 1**).

## Discussion

It has been shown that *S. japonicum* can infect more than 40 species of mammals, including mice, rabbit, goat, and yellow cattle, which are susceptible to infection, whereas other species such as rat, pig, and water buffalo are less susceptible, and the decreased susceptibility is indicated by the smaller worm size, fewer eggs laid, and low developmental rate (He et al., 2001; Yang et al., 2012b). A previous study from our lab has shown that the development of schistosomes (considering worm length, worm recovery rate, and ultrastructural structure) was significantly better than that in water buffaloes, and the liver pathological damage caused by schistosome infections in yellow cattle was more severe than that of water buffaloes (Yang et al., 2012b). In the present study, the average worm recovery rate from water buffalo was also significantly lower than that from yellow cattle. These results suggest that water buffaloes are less susceptible to *S. japonicum* infection than yellow cattle. Our group also compared the transcriptional profiles of adult schistosomes infecting yellow cattle and water buffalo and the results revealed that several genes involved in transcription, transport, lipid metabolism, energy metabolism, nucleotide, and energy and signaling pathways were differentially expressed in worms from these two hosts (Yang et al., 2012a). In this study, we compared the protein expression profiles of adult worms recovered from yellow cattle and water buffalo using iTRAQ. The iTRAQ technique in combination with LC-MS/MS is an effective method for investigating changes in protein levels and provides a more uniform coverage of proteins with unique physical properties (high/low hydrophobicity, *pI*, and *Mw*) than traditional 2-DE. The aim of this study was to elucidate the differences between adult *S. japonicum* infecting yellow cattle and water buffalo at the proteome level. The findings may provide valuable information for better understanding the interplay between *S. japonicum* and its natural hosts. A total of 131 DEPs were identified, of which 46 proteins were upregulated and 85 proteins were downregulated. The results were further confirmed by Western blot analysis and qRT-PCR. The bioinformatics analysis revealed that the identified DEPs were mainly involved in the protein synthesis, transcription regulation, protein proteolysis, cytoskeletal structure and oxidative stress response processes.

Ribosomes synthesize proteins that sustain the growth of biological organisms and are assembled from both ribosomal proteins and rRNA (Meyuhas, 2000). Approximately 80 different ribosomal proteins have been described in eukaryotic cells. Any loss of core ribosomal proteins or any defect in ribosomal components can change gene expression patterns (Thomas, 2000; Kondrashov et al., 2011). Our results indicated that the ribosomal pathway was significantly enriched among the DEPs and a large number of ribosome-associated proteins, including 40S ribosomal protein S24, ribosomal protein L29, ribosomal protein L35, and U3 small nucleolar RNA-associated protein 14, were downregulated in adult schistosomes recovered from water buffalo. This result may explain why schistosomes grow and develop better in yellow cattle than in water buffalo, as ribosomes are responsible for protein synthesis, and the expression of ribosomal proteins is higher in rapidly dividing cells (Pearson and Haber, 1980; Ju and Warner, 1994). A previous study revealed that the pairing of female and male schistosomula triggered a particular ribosomal protein expression profile, and more ribosomal genes or proteins were upregulated in schistosomula from double-sex infections than those from single-sex infections (Sun et al., 2015). We have reported that schistosomes from water buffalo present more pairing retardation than worms from yellow cattle (Yang et al., 2012b). This result suggests that the difference in the expression of ribosomal proteins may affect the development and even the paring of schistosomes in natural final hosts.

Spliceosomes are responsible for splicing the pre-mRNA, which is transcribed from DNA and has exons and introns. After that, the introns are spliced and the remaining exons are joined together to form a mature mRNA, which instructs to build a protein (de Longevialle et al., 2010). RNA splicing is an important posttranscriptional event and is crucial for the regulation of gene expression. The spliceosome consists of five small nuclear ribonucleoprotein particles (U1, U2, U4, U5, and U6 snRNPs) and non-snRNP proteins (Will and Luhrmann, 2011). The association of U1 snRNP with the 5′ splice site is an early event in the spliceosomal pathway and the base-pairing of U1 snRNA to the 5′ splice site occurs by the interaction between pre-mRNA and U1 small nuclear ribonucleoprotein C (U1-C) (Du and Rosbash, 2002; Wahl et al., 2009). The small nuclear ribonucleoprotein F belongs to the pre-mRNA-binding protein family and is involved in the alternative splicing of various genes (Han et al., 2010; Decorsiere et al., 2011; Talukdar et al., 2011). In this study, U1 small nuclear ribonucleoprotein C, small nuclear ribonucleoprotein F, and other spliceosome-associated proteins were downregulated in adult worms from water buffalo, suggesting that the lower expression of these spliceosome-related molecules might affect transcriptional processes in schistosomes infecting water buffaloes and lead to the poor development of the worms.

Cyclophilins have been found in cells from all studied organisms and have peptidyl-prolyl *cis–trans* isomerase activity (Wang and Heitman, 2005). Parasite-encoded cyclophilin proteins act as molecular chaperones by affecting protein folding and assembly and also contribute to RNA splicing (Bell et al., 2006). Previous studies showed that *Sj*Cyclophilin A was an important regulator of schistosome growth in the host environment and played a role in the host-parasite interplay (Han et al., 2012; Li et al., 2013). Histone proteins and DNA are packaged into nucleosomes and form a macromolecular complex known as chromatin. Moreover, the biogenesis of histones is tightly coupled to DNA replication (Anderson et al., 2012). In schistosomes, histone modifications, including acetylation, methylation, phosphorylation, ubiquitinylation, and sumoylation (Liu, 2016), have been reported to play critical roles in DNA repair, chromatin remodeling, and the transcriptional regulation of gene expression (Hong et al., 2016). The histone H4 is a core histone. The downregulation of cyclophilin A and histone H4 observed in the present study may affect transcription and translation in schistosomes infecting water buffaloes.

Cytoskeletal proteins play an important role in the maintenance of cell movement and cell morphology. Myosins are a critical component of the cytoskeleton and bind actin to form microfilaments (Reymann et al., 2012). Tropomyosin is best known for its role in the regulation of the contractile system in muscle and an important cytoskeletal component of non-muscle cells (Gunning et al., 2015). The movement of schistosomes is achieved through good muscular arrangements that are mainly performed by microfilaments and microtubules (Mair et al., 2003). A previous study showed that actin was downregulated in schistosomula from less susceptible rats and resistant *M. fortis* voles compared with susceptible mice (Hong et al., 2011). In the present study, several myosin-related proteins and tropomyosin proteins were downregulated in adult worms from water buffalo, which is consistent with the poor surface topography and internal structures at the ultrastructural level (Yang et al., 2012a). We hypothesize that this process may limit the development of muscle systems and in turn limit the growth of worms. In contrast, the results of iTRAQ and Western blotting indicated that β-actin levels were not significantly different between the two groups, suggesting that the growth and development of schistosomes in natural host animals is different from those of rodents.

The STRING analysis reveals the relations hidden behind the changes of protein levels by means of a computer-assisted analytical approach (Xu et al., 2017). In this study, the protein–protein relationships within the DEPs were also examined using STRING. It was of note that the downregulated proteins formed three subsets of protein interaction networks: protein synthesis, transcription regulation, and cytoskeleton-associated proteins. This result suggests that the downregulation of these proteins maybe affect the growth and development of schistosomes. Furthermore, there was no significant interactions among the upregulated proteins and this result needs to be further investigated.

Lysosomes are lytic vacuoles that contain acid hydrolase enzymes involved in the cleavage of waste materials, including cellular debris and exogenous proteins (Turk et al., 2002). Proteolysis in lysosomes is an important protein degradation pathway. The tetraspanin protein 2, also known as CD63, is a lysosomal membrane protein that plays an important role in the formation of phagosomes (Pols and Klumperman, 2009; Reymann et al., 2012). Cathepsin L is a lysosomal endopeptidase involved in terminal protein degradation, and the upregulation of cathepsin L has been reported in a wide range of malignancies (Dou and Carruthers, 2011; Sudhan and Siemann, 2015). The lysosomal protein saposin B is critical for lipid degradation metabolism (Huta et al., 2016), and several proteins containing the saposin B domain were reported to act as lysosome carrier proteins that bind sphingolipids to facilitate their degradation in *Schistosome mansoni* (Hall et al., 2011). Autophagy is the primary pathway for the degradation of dysfunctional macromolecules and lysosomal processes (Hong et al., 2016). The water buffalo can develop the phenomenon of self-cure of *S. japonicum* infection and the worms usually do not grow in the animals for 1–2 years post infection (Angeles et al., 2015). The upregulation of three lysosome-associated proteins—tetraspanin protein 2, cathepsin L, and saposin B domain-containing protein—in schistosomes from water buffalo might be partially correlated with this phenomenon.

Reactive oxygen species (ROS) are found in normal living organisms where they are constantly being produced under the oxidative stress caused by toxic heavy metals, heat shock, inflammation, ionizing irradiation, immune responses and environmental stimuli (Yu, 1994; Pushpamali et al., 2008). In schistosomes, ROS has been reported to be highly toxic, and parasites have developed antioxidant defense systems to protect themselves against ROS (Kazura et al., 1981; Hong et al., 2013). Thioredoxin peroxidase-1 (TPx-1) belongs to the family of thioredoxin peroxidases and is a major contributor to the detoxification of hydrogen peroxide in helminth parasites (Kumagai et al., 2006). Protein disulfide isomerase (PDI) belongs to the thioredoxin (Trx) superfamily and contains several Trx domains with redox active thiol/disulfide motifs (Gilbert, 1998). *Sj*PDI may protect schistosomes from the oxidative damage induced by ROS, which is produced by host immune cells during oxygen metabolism (Cao et al., 2014). Glutathione *S*-transferases (GSTs) are a family of isoenzymes involved in the detoxification of potentially harmful electrophilic compounds by binding a range of hydrophobic ligands or by conjugation to glutathione (GSH) (Luo et al., 2009). There are 26 kDa and 28 kDa two isoenzyme GST molecules exist in schistosome. The 26-kDa GST of *S. japonicum* has been well characterized and several studies have demonstrated that r*Sj*26GST could induce a strong protection against *S. japonicum* infection in mice, pig, sheep, water buffalo, and other animals (Wu et al., 2005). In the present study, *Sj*TPx-1, *Sj*PDI, and *Sj*26GST were significantly upregulated in schistosomes from water buffaloes, which were less susceptible hosts. We hypothesize that the overexpression of the three proteins may be necessary for the parasite to adapt to the host environment and enable survival and development in the less susceptible host.

We observed that the function of some identified DEPs was unknown or unannotated. Although the genome of *S. japonicum* was sequenced and assembled in 2009 (Zhou et al., 2009), the annotation of coding genes and proteins is still being developed. Therefore, the mechanisms involved in the susceptibility of natural hosts to schistosomes at the protein level need to be better explained with the gradual improvement of genome annotation.

Western blotting is a technique suitable to confirm the iTRAQ results but is limited by the availability of only a few specific antibodies. For this reason, qRT-PCR was carried out to verify the reliability of the iTRAQ data at the transcriptional level. The results revealed that the protein expression pattern of two selected DEPs did not coincide with the transcriptional expression pattern. This discrepancy may be due to the higher sensitivity of iTRAQ compared to qRT-PCR or posttranscriptional regulation (Vogel and Marcotte, 2012; Lv et al., 2017). Consistent with the results in this study, spliceosome and a mRNA surveillance pathway were significantly enriched among the DEPs. In addition, miRNAs play important roles in gene regulation and usually bind to the 3′UTR of their target mRNAs and cause mRNA degradation or translational inhibition (Liu, 2016). We previously found that miRNAs were differentially expressed between schistosomes from susceptible and less susceptible rodent hosts (Han et al., 2015a,b). This mechanism may also occur in worms from different natural hosts.

The DEPs information from the current study is fundamental to understand the different susceptibility to *S. japonicum* infection in water buffalo and yellow cattle. In the next work, we will silence some genes of *S. japonicum* in the *in vitro* cultural environment by RNAi according to the data of this study. We expect to further understand the host–parasite relationships in water buffalo and yellow cattle and gain useful information for screening bovine schistosome vaccine candidates. If the veterinary vaccine can be successfully developed, the contributions to the elimination of *S. japonicum* in China might be amazing.

## Conclusion

In the present study, we used iTRAQ to identify DEPs in adult schistosomes from a *S. japonicum-*susceptible natural host (yellow cattle) and a less-susceptible natural host (water buffalo). The bioinformatics analysis indicated that some DEPs might have affected the survival and development of schistosomes. The identification of these proteins provides a new basis to understand the developmental mechanism of schistosomes and the interplay between schistosomes and their natural hosts.

## Author Contributions

JjL and GL conceived and designed the study. QZ, ZF, YH, XY, QH, KL, HL, XD, CZ, and JmL performed the experiments. QZ, ZF, and YH analyzed the data. QZ, JjL, and GL wrote the paper. QZ and YH revised the manuscript. All authors read and approved the final manuscript.

## Conflict of Interest Statement

The authors declare that the research was conducted in the absence of any commercial or financial relationships that could be construed as a potential conflict of interest.
