# Supplementary material for: iTRAQ-Based Comparative Proteomic Analysis of Adult Schistosoma japonicum from Water Buffalo and Yellow Cattle
Source: Front Microbiol. 2018 Feb 6;9:99. doi: 10.3389/fmicb.2018.00099 (PMC5808103; doi:10.3389/fmicb.2018.00099)
Supplement: Supplementary file 1 [file Table_1.DOCX]

**Supplemental Table 1.** Perfusion recovery of adult worms from water buffalo and yellow cattle (Mean ± SD)

| Species | Adult worm recovery (%) |
| --- | --- |
| Water buffalo  Yellow cattle | 10.8 ± 3.3^*^  68.8 ± 10.8 |

* *P* <0.05 for significantly different when compared to yellow cattle group.
