# Supplementary material for: iTRAQ-Based Comparative Proteomic Analysis of Adult Schistosoma japonicum from Water Buffalo and Yellow Cattle
Source: Front Microbiol. 2018 Feb 6;9:99. doi: 10.3389/fmicb.2018.00099 (PMC5808103; doi:10.3389/fmicb.2018.00099)
Supplement: Supplementary file 5 [file Image_1.PDF]

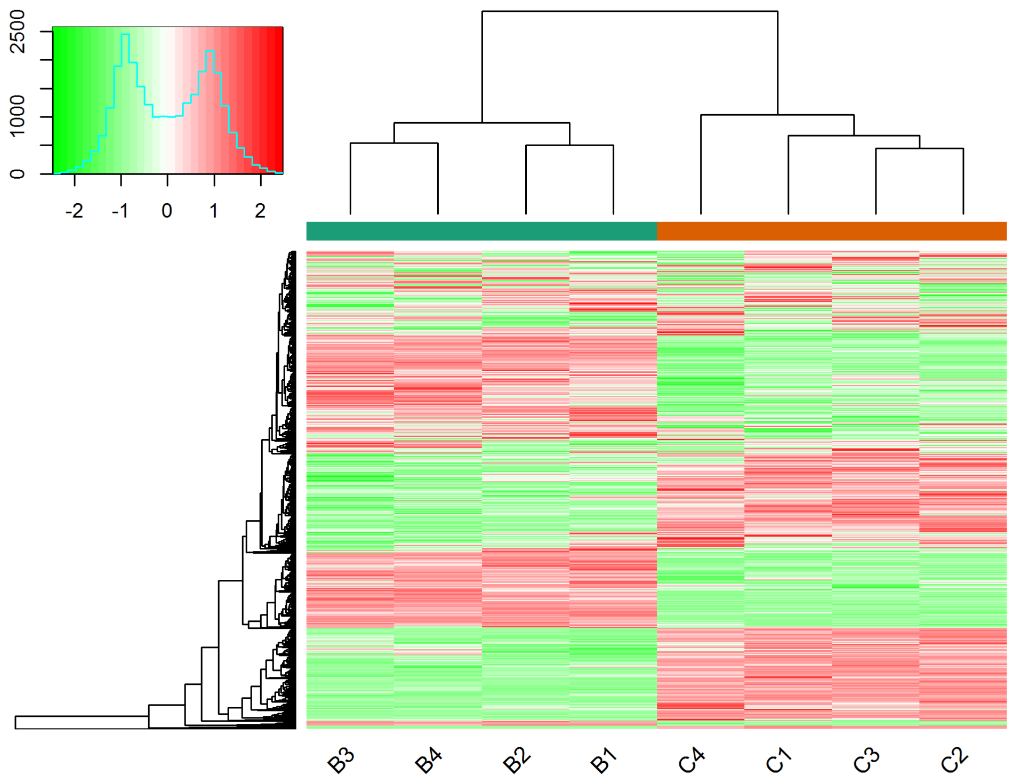

Supplementary figure 1. Cluster analysis for the proteins of adult schistosome from water buffalo and yellow cattle. B1, B2, B3 and B4 indicate the water buffalo group. C1, C2, C3 and C4 indicate the yellow cattle group.
