# Supplementary material for: iTRAQ-Based Comparative Proteomic Analysis of Adult Schistosoma japonicum from Water Buffalo and Yellow Cattle
Source: Front Microbiol. 2018 Feb 6;9:99. doi: 10.3389/fmicb.2018.00099 (PMC5808103; doi:10.3389/fmicb.2018.00099)
Supplement: Supplementary file 6 [file Image_2.PDF]

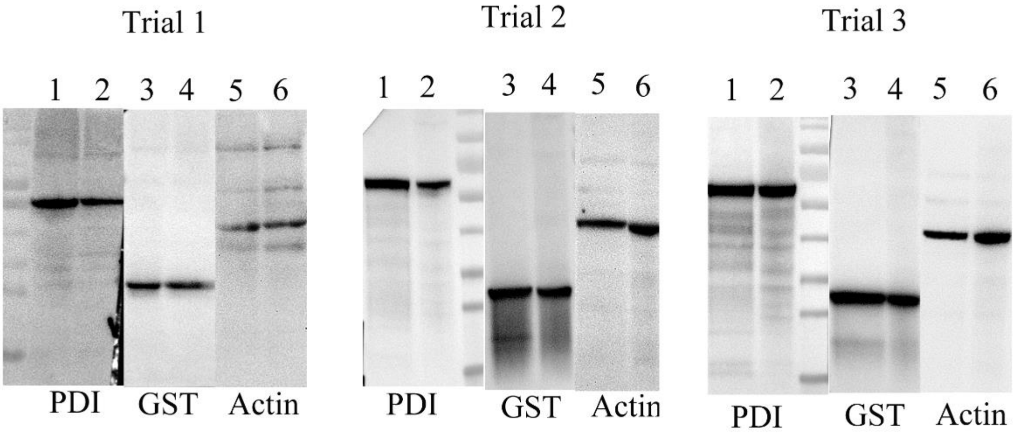

Supplementary figure 2. Confirmation of differentially expressed proteins by Western blotting. Lane 1, *Sj*PDI of water buffalo group. Lane 2, *Sj*PDI of yellow cattle group. Lane 3, *Sj*GST of water buffalo group. Lane 4, *Sj*GST of yellow cattle group. Lane 5, actin of water buffalo group. Lane 6, actin of yellow cattle group.
